# Molecular characterization and transcriptional regulation of two types of H^+^-pyrophosphatases in the scuticociliate parasite *Philasterides dicentrarchi*

**DOI:** 10.1038/s41598-021-88102-0

**Published:** 2021-04-19

**Authors:** I. Folgueira, J. Lamas, R. A. Sueiro, J. M. Leiro

**Affiliations:** 1 Department of Microbiology and Parasitology, Institute of Research on Chemical and Biological Analysis (IAQBUS), University of Santiago de Compostela, 15782 Santiago de Compostela, Spain; 2 Department of Fundamental Biology, Institute of Aquaculture, University of Santiago de Compostela, 15782 Santiago de Compostela, Spain

**Keywords:** Biochemistry, Microbiology, Molecular biology, Diseases

## Abstract

Proton-translocating inorganic pyrophosphatases (H^+^-PPases) are an ancient family of membrane bound enzymes that couple pyrophosphate (PPi) hydrolysis to H^+^ translocation across membranes. In this study, we conducted a molecular characterization of two isoenzymes (PdVP1 and PdVP2) located in respectively the alveolar sacs and in the membranes of the intracellular vacuoles of a scuticociliate parasite (*Philasterides dicentrarchi*) of farmed turbot. We analyzed the genetic expression of the isoenzymes after administration of antiparasitic drugs and after infection in the host. PdVP1 and PdVP2 are encoded by two genes of 2485 and 3069 bp, which respectively contain 3 and 11 exons and express proteins of 746 and 810 aa of molecular mass 78.9 and 87.6 kDa. Topological predictions from isoenzyme sequences indicate the formation of thirteen transmembrane regions (TMRs) for PdVP1 and seventeen TMRs for PdVP2. Protein structure modelling indicated that both isoenzymes are homodimeric, with three Mg^2+^ binding sites and an additional K^+^ binding site in PdVP2. The levels of identity and similarity between the isoenzyme sequences are respectively 33.5 and 51.2%. The molecular weights of the native proteins are 158 kDa (PdVP1) and 178 kDa (PdVP2). The isoenzyme sequences are derived from paralogous genes that form a monophyletic grouping with other ciliate species. Genetic expression of the isoenzymes is closely related to the acidification of alveolar sacs (PdVP1) and intracellular vacuoles (PdVP2): antiparasitic drugs inhibit transcription, while infection increases transcription of both isoenzymes. The study findings show that *P. dicentrarchi* possesses two isoenzymes with H^+^-PPase activity which are located in acidophilic cell compartment membranes and which are activated during infection in the host and are sensitive to antiparasitic drugs. The findings open the way to using molecular modelling to design drugs for the treatment of scuticociliatosis.

## Introduction

H^+^-translocating pyrophosphatases (H^+^-PPases) belong to the family of membrane integral hydrophobic proteins known to couple the energy obtained from the hydrolysis of pyrophosphosphate (PPi) into two orthophosphate (Pi) molecules as the driving force for H^+^ movement across biological membranes^1–5^. From a functional point of view, H^+^-PPases are grouped with the soluble pyrophosphatases (sPPases), with which they can cooperate to maintain PPi homeostasis in the cytosol of plant cells^6,7^. H^+^-PPase has two main physiological roles in plants: hydrolysis of PPi in the cytosol and acidification of vacuoles^7^. sPPases have been identified in both prokaryotes and eukaryotes (including animals, plants and fungi)^8–10^. The H^+^-PPases are ubiquitous in plants and have also been identified in some parasitic protozoa, archaea and bacteria; however, so far they have not been described in either fungi or animals^2,4,11–24^. In plants, H^+^-PPases are divided into types I and II, which correspond to two isoforms that differ according to their K^+^ dependence and Ca^2+^ sensitivity, although both types require Mg^2+^ activity as a cofactor^3,13,17^. The existence of H^+^-PPases in vacuoles (tonoplast), Golgi apparatus, plasma membrane, mitochondria and chloroplast of plants has also been demonstrated, although the corresponding genes have not always been identified^11,25–31^. The type I H^+^-PPase (VP1) is mainly located in the vacuole membrane^32^, and increased concentrations have been associated with a PPi-scavenging role^33^, while type II H^+^-PPase (VP2) is preferably located in the Golgi apparatus^29,34^. Some protozoa, such as *Trypanosoma brucei* and *Plasmodium falciparum*, have small acidic organelles (acidocalcisomes) that accumulate orthophosphate (Pi), PPi and polyphosphate (polyP). The acidocalcisomes play an essential role in regulating pH, osmotic homeostasis, calcium signalling and possibly in autophagy^35,36^, and have vacuolar proton pyrophosphatases (V-H^+^-PPase, or VP1) on their membranes^21,37,38^.

*Philasterides dicentrarchi*^39^ is a cosmopolitan free-living marine amphizoic scuticociliate that can transform into a parasite that causes a disease called scuticociliatosis, which is particularly severe in farmed flatfish^40,41^, such as turbot^42^. Like other scuticociliates*, P. dicentrarchi* is a microaerophilic, euryhaline organism capable of surviving conditions of hypoxia and hyposalinity^43–46^. In a previous study, we characterized, for the first time at molecular and functional levels, an H^+^-PPase in *P. dicentrarchi* located in the membrane of the vacuoles and alveolar sacs ^22^. Based on the presence of this enzyme in phagocytic vacuoles and alveolar sacs, it has been suggested that H^+^-PPase in *P. dicentrarchi* may possess, like some plant species^3^, two isoforms that are probably generated by alternative splicing^47^.

In the present study, we used next-generation sequencing (NGS) to verify the presence of two sequences corresponding to two complete genes and their corresponding transcripts belonging to the H^+^-PPase of the scuticociliate parasite *P. dicentrarchi*. We also showed that H^+^-PPase activity in *P. dicentrarchi* is associated with two isoenzymes previously characterized at the molecular level. We determined the cellular location of the isoenzymes and analysed the transcriptional regulation of the isoenzymes during infection in turbot and after the administration of physiological stimuli and antiparasitic drugs that modulate enzymatic activity.

## Materials and methods

### Experimental animals and ethics statement

In some experiments, we used juvenile turbot, *Scophthalmus maximus* (L.), weighing 50 g. The specimens were obtained from a commercial fish farm in Galicia (Northwest Spain). Once in the experimental aquarium at the University of Santiago de Compostela, the fish were placed in seawater tanks of about 200 L capacity, which were connected to a recirculating water system maintained at a temperature of 16ºC, with constant aeration. The fish were subjected to a 12L: 12D photoperiod and fed with commercial feed (Skretting, Burgos, Spain). The fish were acclimatized to the aquarium conditions for at least 2 weeks before the experiments were carried out.

Female Crl: CD1 (ICR) mice (Charles River, USA) of average weight 25 g, supplied by the Central Animal Husbandry of the University of Santiago de Compostela, were used to obtain the antibodies used in the immunoassays.

All animal experiments and protocols were conducted in accordance with Spanish and European Legislation (R.D. 53/2013 and Council Directive 2010/63/EU) and approved by the Institutional Animal Care and Use Committee of the University of Santiago de Compostela (Spain). In addition, it is reported that all animal experiments conducted in this study were conducted in accordance with guidelines ARRIVE (https://arriveguidelines.org). When required in the experiments, the animals were anaesthetized with isoflurane (mice) or 100 mg / L of tricaine methane sulphonate (MSS-222) (turbot) and finally euthanized by decapitation (mice) or by overdose of anaesthesia (turbot).

### Ciliate culture and experimental infections

We used the I1 isolate obtained from ascitic fluid from naturally infected turbot^42^. Ciliates were cultured in Leibovitz L-15 medium supplemented with 10% inactivated bovine serum, lipids (lecithin and Tween 80), nucleotides and glucose, under the culture conditions described by Iglesias et al.^43^.

Experimental infections in turbot were performed by intraperitoneal injection in turbot with 5 × 10^5^ ciliates, as previously described^48^.

### Sequencing of the genome and transcriptome

For analysis of the *P. dicentrarchi* genome and transcriptome, trophonts (10^7^) were concentrated by centrifugation, frozen in liquid nitrogen and sent on dry ice to Future Genomic Technologies (Leiden, Netherlands). For sequencing of the complete genome of the ciliate, a combination of short reading sequencing (Illumina technology) and long reading sequencing (Nanopore technology) was used (Oxford Nanopore Technologies)^49^. For de novo assembly of the parasite genome, the data sets were combined using TULIP software, v0.4^50^. For sequencing of the *P. dicentrarchi* transcriptome, we followed the RNAseq procedure as previously described^51,52^.

### Production of recombinant protein in yeast cells and peptide synthesis

To produce the recombinant protein, we selected a region of the mRNA sequence corresponding to H^+^ PPase type I (rPdVP1) of *P. dicentrarchi*^22^ which we then translated following the standard genetic code (see Fig. 5) using the protocol as previously described^47^. Briefly, *P. dicentrarchi* RNA was purified using the NucleoSpin RNA kit (Macherey–Nagel, Germany), and cDNA synthesis was performed with a reaction mixture containing 1.25 µM random hexamer primers (Promega), 250 µM of a mixture of deoxynucleoside triphosphates (dNTPs), 10 mM of dithiothreitol (DTT), 20 U of RNase inhibitor, 2·5 mm MgCl_2_, 200 U of MMLV (Moloney murine leukaemia virus reverse transcriptase (Promega) in 30 mm Tris and 20 mm KCl (pH 8·3) and 2 µg of *P. dicentrarchi* RNA. PCR was performed on the cDNA using the following pair of primers designed and optimized for expression in the yeast *Saccharomyces cerevisiae*: 5′ AAAGAAGAAGGGGTACCTTTGGATAAAAGAattgatgtcaacgccccctt and 3′/5′- TGGGACGCTCGACGGATCAGCGGCCGCTTAGTGGTGGTGGTGGTGGTGgggaccagaggtatctttta-3′. The PCR product obtained contained a hybridization region with the yeast YEpFLAG-1 (Eastman Kodak Company) and a poly-His tail. The product was cloned in YEpFLAG-1 (Eastman Kodak Company) yeast expression vector, digested with *EcoRI* and *SalI* (Takara) and used to transform *Saccharomyces cerevisiae* cells (strain BJ 3505). Positive colonies were selected on complete, tryptophan-free medium (CM-Trp) containing glucose (20 g L^−1^), Yeast Nitrogen Base medium without amino acids (Sigma-Aldrich), supplemented with adenine (40 mg L^−1^) and amino acids (histidine, leucine, tyrosine, 40 mg L^−1^ each; arginine, methionine, threonine 10 mg L^−1^ each; isoleucine and phenylalanine 60 mg L^−1^ each and lysine 40 mg L^−1^). The recombinant rPdVP1 protein was extracted from transformed *S. cerevisiae* cultures, which were incubated for 72 h in modified yeast peptone, high stability expression medium containing 1% glucose, 3% glycerol, 1% yeast extract and 8% peptone, at 30 °C, by immobilized metal affinity chromatography (IMAC) and using a pre-charged Ni-Sepharose Histrap column, as previously described^22^.

The HKAAVIGDTIGDPLK peptide (HK peptide) corresponding to PdVP1 was chemically synthesized by adding a cysteine amino acid to enable conjugation to the carrier keyhole-limpet haemocyanin (KLH-HK) protein (ProteoGenix, France).

### Immunization and serum preparation

Crl: CD1 (ICR) mice were immunized intraperitoneally with 200 µL of a 1:1 (v/v) mixture of complete Freund's adjuvant and a solution containing 500 µg of the truncated recombinant PdVP1 protein (rPdVP1, Fig. 3A) or with 500 µg of synthetic peptide HK (PAB_HK_ motif) conjugated to KLH (KLH-HK) of PdVP1 (Fig. 3A). Fifteen and thirty days after the first immunization, the mice were injected with the same dose of rPdVP1 and KLH-HK emulsified in incomplete Freund's adjuvant. Seven days after the last immunization, the mice were bled by decapitation. The blood was allowed to clot at 4 °C overnight, and the serum was obtained by centrifugation at 2000 xg for 10 min and mixed with glycerol 1: 1 (v / v) and stored at -20ºC until use.

### Western blotting

A sample of lysed ciliates prepared as previously described^53^, treated under reducing conditions (after the addition of 200 mM of dithiothreitol-DTT) or under non-reducing conditions (without the addition of DTT), was separated by 12.5% linear sodium dodecyl sulphate polyacrylamide gel electrophoresis (SDS-PAGE), using pre-stained molecular weight (MW) markers as molecular size standards^53^. The separated proteins were electro-transferred to membranes of 0.45 µm PVDF (Hybond-P, GE Healthcare) in a semi-dry transfer system (Trans-Blot SD, Biorad), as previously described^54^. The membranes were immunoblotted with a 1: 100 dilution of the anti-rPdVP1 (α-rPdVP1) and anti-KLH-HK (α-KLH-HK) antibodies and then with a polyclonal peroxidase-conjugated rabbit anti-mouse antibody (Dakopatts, Denmark) at 1: 800 dilution. The blots were stained by adding a chromogenic enzyme substrate solution consisting of 0.003% H_2_O_2_ and 0.06% 3,30-diaminobenzidine tetrahydrochloride with 0.03% NiCl_2_ (DAB/NiCl_2_, Sigma, USA)^51^.

### Indirect immunofluorescence (IIF)

For immunolocalization of two isoenzymes (PdVP1 and PdVP2) in trophonts, an IIF assay was performed as previously described^22^. Briefly, 10^6^ ciliates were fixed in a solution of 4% formaldehyde in PBS, permeabilized in a solution containing 0.1% Triton X-100 in PBS and blocked with a solution of 1% BSA. The ciliates were then incubated with a mouse antiserum containing α-rPdVP1 and α-KLH-HK antibodies diluted 1:100 in PBS and with a secondary polyclonal rabbit anti-mouse immunoglobulin conjugated with fluorescein isothiocyanate (FITC; DAKO, Denmark) and diluted 1:1,000. The ciliates were then visualized by fluorescence microscopy (Zeiss Axioplan, Germany).

### Acridine orange staining

PdVP activity in *P. dicentrarchi* trophozoites was detected with the fluorimetric stain acridine orange, a pH sensitive fluorescent cationic dye which accumulates in acidic compartments and which is commonly used as an indicator of transmembrane pH difference in permeabilized ciliates^55,56^. The protocol used was previously described by Mallo et al.^57^. Briefly, the ciliates were permeabilized with 6.6 μM of digitoninin and washed by centrifuging twice with PBS. The ciliates were then resuspended in PBS buffer containing 1.3 mM MgSO_4_ and 3 mM acridine orange. Tris-PPi (1 mM) was added immediately at the start of the experiment, and the ciliates were incubated for 60 min. Staining was observed 10, 30 and 60 min after PPi addition in a fluorescence microscope (Zeiss Axioplan, Germany) with an excitation BP 546 nm dichroic mirror filter and FT 580 nm LP emission 590 nm filter.

### Reverse transcriptase-quantitative polymerase chain reaction (RT-qPCR)

The RT-qPCR technique was performed as previously described^51^. The total RNA from 10^6^ trophozoites/mL incubated with 1 mM Tris-PPi (for 0, 30 and 60 min); with 100 µM of resveratrol (RESV), artemisinin (ART), chloroquine (CLQ) and with CaCl_2_ (1 mM) for 2 h; or obtained of intraperitoneally infected turbot 2 h after infection, was isolated with a Nucleospin RNA isolation kit (Macherey–Nagel) according to the manufacturer’s instructions. The cDNA synthesis (RT reaction) was conducted as described earlier, and the quantitative polymerase chain reaction (qPCR) was performed with the primer pairs FPdVP1/RPdVP1 (5′ CGTCGGATTACTCTGGGCTA/AAGAAGGCGTTGGATCCTCT) (PdPV1), FPVP2/RPVP2 (5′ TGGGATTCGTTTTCTTTCCTT/CATTTCTCCTGTTCCTGTTTCTTT 3′) (PdPV2) and *P. dicentrarchi* elongation factor 1-alpha gene (EF-1α) (GenBank accession KF952262) forward/reverse primer pair (FEF1A/REF1A: 5′-TCG CTC CTT CTT GCA TCG TT-3′/ 5′-TCT GGC TGG GTC GTT TTT GT-3′) as a housekeeping gene. For the qPCR, PowerUP SYBR™ Gren Master Mix (Applied Biosystems) was used according to the manufacturer’s instructions, with the following thermocycling conditions: 95 °C for 5 min, followed by 40 cycles at 95 °C for 10 s and 60 °C for 30 s, ending with melting-curve analysis at 95 °C for 15 s, 55 °C for 15 s and 95 °C for 15 s, carried out in a StepOnePlus Real Time System (Thermo Fisher Scientific). Transcription levels were determined using the 2^−ΔΔCt^ method^58^ in accordance with the MIQUE guidelines^59^, as follows: the Ct value for the reference gene (EF-1α) was subtracted from the Ct value of the FdVP1 and FdVP2 genes when the reaction occurred with 100% efficiency and was in the exponential phase, thus producing ΔCt; the value obtained after the specific treatment was then subtracted from ΔCt, to yield the -ΔΔCt used as the exponent of 2 in the equation and that represents represents the fold change of the treated target gene relative to the control.

### Bioinformatic and statistical analysis

Functional analysis of proteins and classification into different families -in order to predict the domains and important sites- was carried out using InterPro software^60^. The transmembrane topology and location of signal peptide cleavage sites in amino acid (aa) sequences were predicted using the Phobius program^61^. Proteoforms were visualized and the protein topology was integrated using the Protter bioinformatics tool^62^. The physicochemical parameters were predicted for a given protein using the ProtParam tool^63^. The O-ß-GlcNAc attachment sites were predicted using the YingOYang^64^ and OglcNacScan^65^ programs. Sub-cellular localization was predicted using the LocTree3 program^66^. Protein model structures are computed by the SWISS-MODEL server homology modelling pipeline^67^, which relies on ProMod3, an in-house comparative modelling engine based on OpenStructure^68^. The aa sequences were aligned using the Clustal Omega multiple sequence alignment program^69^, and the phylogenetic signal / noise ratio was improved using the TrimAI tool for automated alignment trimming^70^. The phylogenetic trees were constructed using the Maximum Likelihood (ML) method, with the JTT model^71^. Branch support was given with 500 bootstrap replicates^72^ in MEGAX software^73^.

The values shown in the text and figures are means ± SEM of five samples, each tested in triplicate. One-way analysis of variance (ANOVA) was used for comparison of more than two samples, and the Tukey–Kramer test was used for pairwise comparisons. The Student´s t-test was used for comparison of two samples. In all cases, differences were considered significant at *P* < 0.05.

## Results

### Overall structure of H^+^-PPases in *P. dicentrarchi*

**Figure 1:**
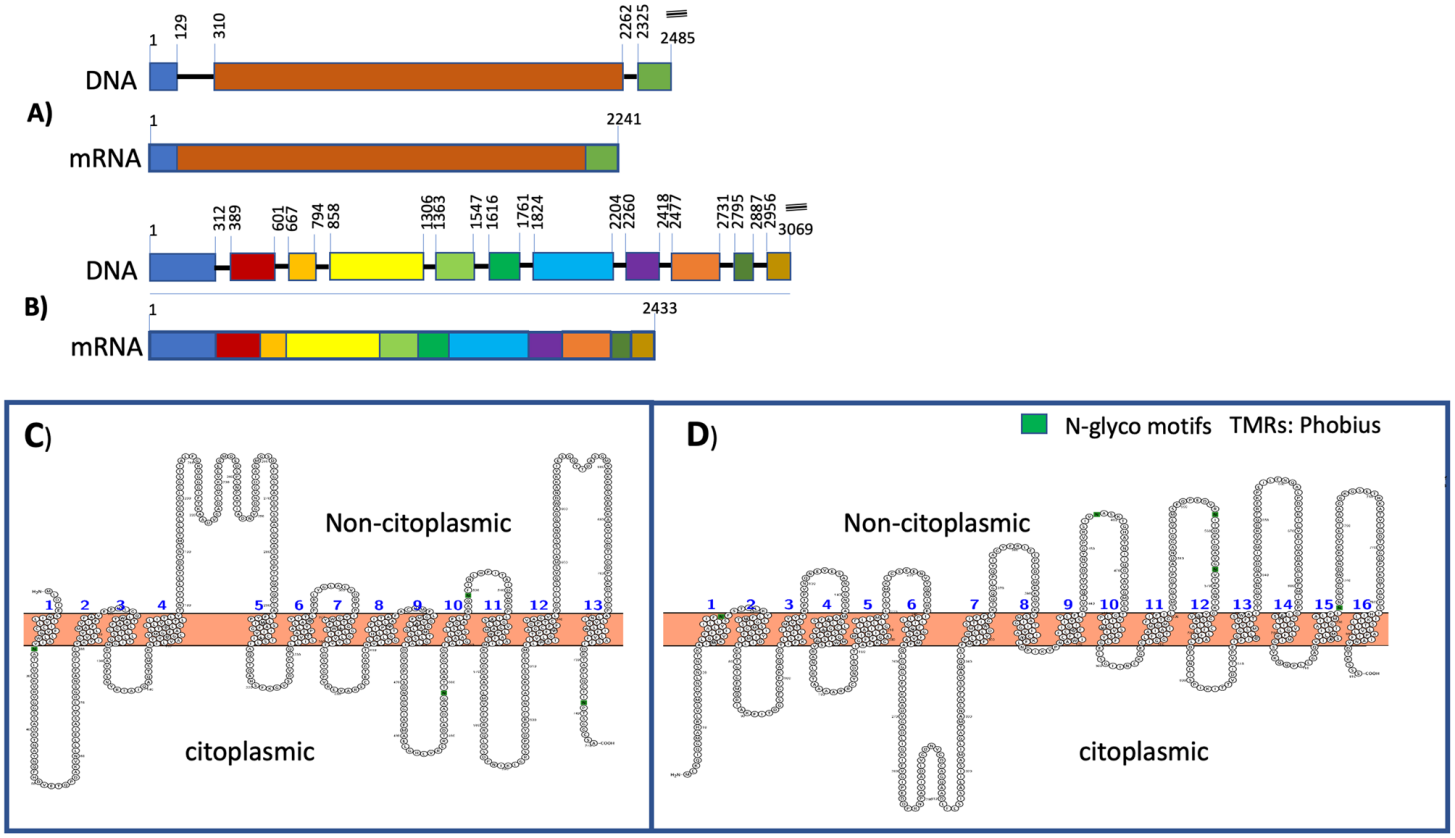
Schematic representation of the genes corresponding to the two isoenzymes (type 1 and 2; PdVP1, **A**; and PdVP2, **B**) of the H + -PPases present in *P. dicentrarchi*, together with their corresponding translation to mRNA. The figure shows the position of the exons (squares) and introns (lines) in the nucleotide sequence. Linear scale = 100 nucleotides. Topology of the amino acid sequence of two H^+^-PPase isoenzymes (PdVP1, **C**; PdVP2, **D**), including the predictions of the transmembrane regions (TMRs) and O-ß-GlcNAc attachment sites. Topological structures were generated using Protter v1.0 (http://wlab.ethz.ch/protter), the predictions of TMRs were generated using the Phobius program (https://www.ebi.ac.uk/Tools/pfa/phobius/) and the O-ß-GlcNAc attachment sites were predicted using the programs YinOYang 1.2 and NetOGlyc 4.0 (http://www.cbs.dtu.dk/services/YinOYang/; http://www.cbs.dtu.dk/services/NetOGlyc/). The box is a schematic representation of the plasma membrane with the extra- and intracellular regions specified.

The sequences corresponding to the isoenzymes of the H^+^-PPases of *P. dicentrarchi* were deposited in the GenBank database with the following access numbers: MN207485, *P. dicentrachi* H^+^-translocating pyrophosphatase gene, complete cds (*P. dicentrarchi* alveolar pyrophosphatase, PdVP1); MN207486, *P. dicentrarchi* H^+^-translocating pyrophosphatase gene, complete cds (*P. dicentrarchi* vacuolar pyrophosphatase, PdVP2); MN193567, *P. dicentrarchi* H^+^ translocating inorganic pyrophosphatase 1 mRNA, complete cds (PdVP1) and MN193568, *P. dicentrarchi* H^+^-translocating inorganic pyrophosphatase 2 mRNA, complete cds (PdVP2). The gene encoding the PdVP1 is of total length 2485 bp and contains 3 exons and 2 introns (Fig. 1A). The coding sequence contains 2241 bp that encode a protein of 746 aa (GenBank Protein accession QIJ96372), of estimated molecular weight 78,947.27 daltons (Da) and a theoretical pI of 5.09. The gene encoding the PdVP2 is of total length 3069 bp and contains 11 exons and 10 introns (Fig. 1B). The coding sequence contains 2433 bp that encode a protein of 810 aa (GenBank Protein Accession QIJ96373) with an estimated molecular weight of 87,596.32 Da and theoretical pI of 5.36. Bioinformatic predictions using the InterPro program, which performs functional protein analysis and family rankings, place the PdVP1 and PdVP2 protein sequences within the family of pyrophosphatase-energized proton pumps. Regarding establishment of the gene ontology (GO) terms, InterPro predicts that both PdVP1 and PdVP2 participate in biological processes of proton transmembrane transport (GO: 1902600), with inorganic diphosphatase activity (GO: 0004427) and pyrophosphatase hydrolysis-driven proton transmembrane transporter activity (GO: 0009678) as the main molecular functions, while in relation to the cellular component, both proteins are associated with the cell membranes (GO: 0016020).

The proteoforms of the aa sequences of the PdVP1 and PdVP2 were visualized using the Protter program, which integrates the prediction of the transmembrane topology carried out by the Phobius program (Figs. 1C,D). Analysis of the topology of the aa sequences indicates that both PdVP1 and PdVP2 lack signal peptide, and PdVP1 forms thirteen transmembrane regions (TMRs) (Fig. 1C), while PdVP2 forms sixteen transmembrane regions (Fig. 1D). In PdVP1, TMRs are generated between aa at positions 6–24, 81–99, 105–127, 148–178, 292–312, 332–351, 366–390, 411–435, 441 -463, 507–525, 545–565, 614–640 and 708–728; seven of the regions have a cytoplasmic location located between aa at positions 25–80, 128–147, 313–331, 391–410, 464–506, 566–613 and 728–746; another seven regions have a non-cytoplasmic location between aa at positions 1–5, 100–104, 179–291, 352–365, 436–440, 526–544 and 641–707 (Fig. 1C). Regarding the sequence of the PdVP2, the Phobius program predicts that the TMRs are generated between aa at positions 25–47, 53–77, 106–124, 144–171, 191–219, 239–257, 343–365, 394 -411, 418–438, 478–496, 508–530, 575–593, 614–633, 685–703, 715–734, 782–804; presenting nine cytoplasmic localization regions located between aa at positions 1–24, 78–105, 172–190, 258–342, 412–417, 497–507, 594–613, 704–714 and 805–810 and eight localization regions non-cytoplasmic located between aa at positions 48–52, 125–143, 220–238, 366–393, 439–477, 531–574, 634–684 and 735–781 (Fig. 1D).

### Protein modelling of H^+^-PPases

**Figure 2:**
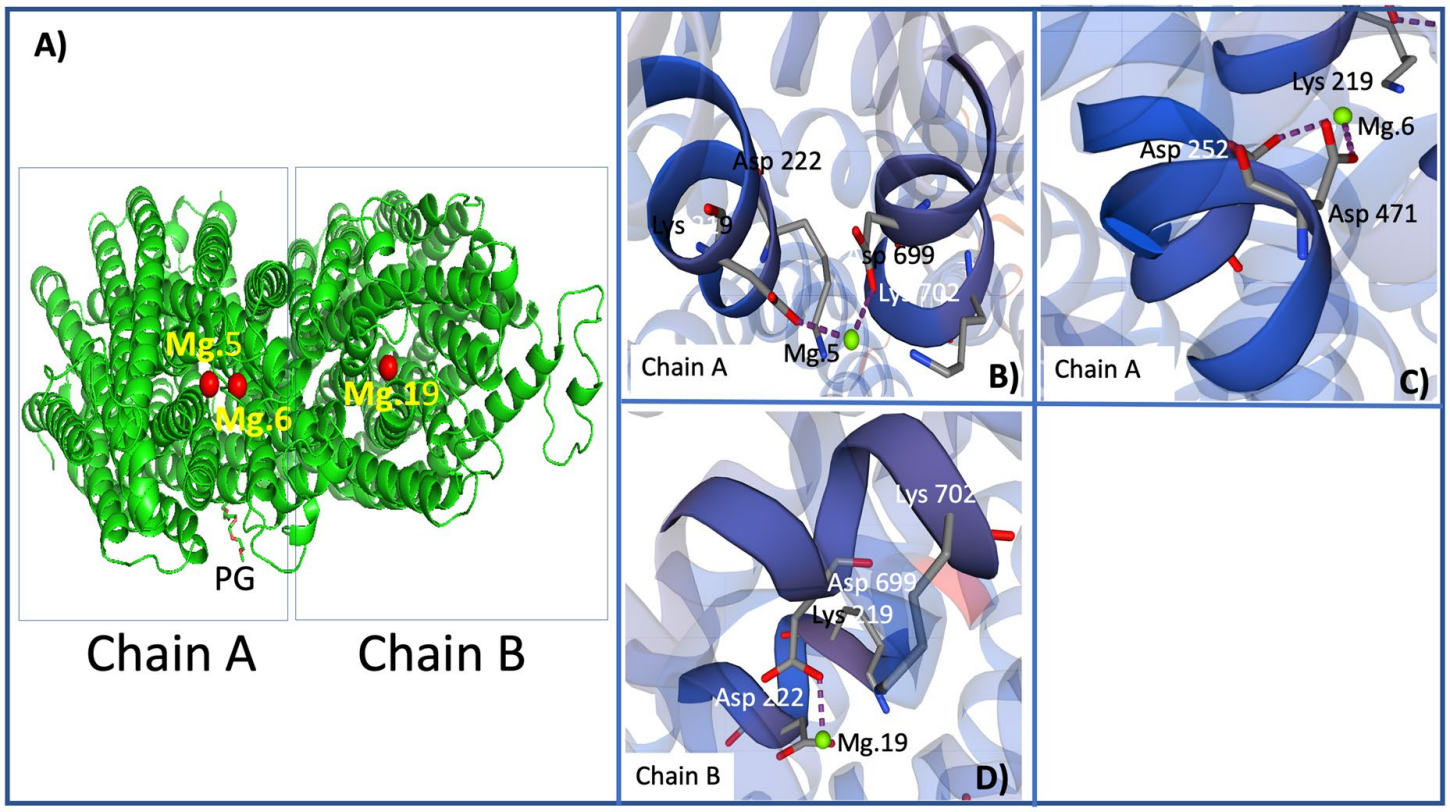
(**A**) Modelling of the three-dimensional structure corresponding to the protein sequence of the isoenzyme PdVP1 of the H^+^-PPase of *P. dicentrarchi*. The homodimeric type oligomeric model obtained from the quaternary structure is included, indicating the existence of two monomeric chains A and B (circles). Mg.5 has four coordination residues and two protein–ligand interactions (PLIPs) in the A chain formed metal complexes (**B**). Mg. 6 has three coordination residues and two PLIP interactions in the A chain forming metal complexes (**C**). Mg.19 has four coordination residues in the B chain protein and two PLIPs (**D**). Templates were obtained by an automated comparative protein modelling server of Swiss-Model (URL: https://swissmodel.expasy.org/).

**Figure 3:**
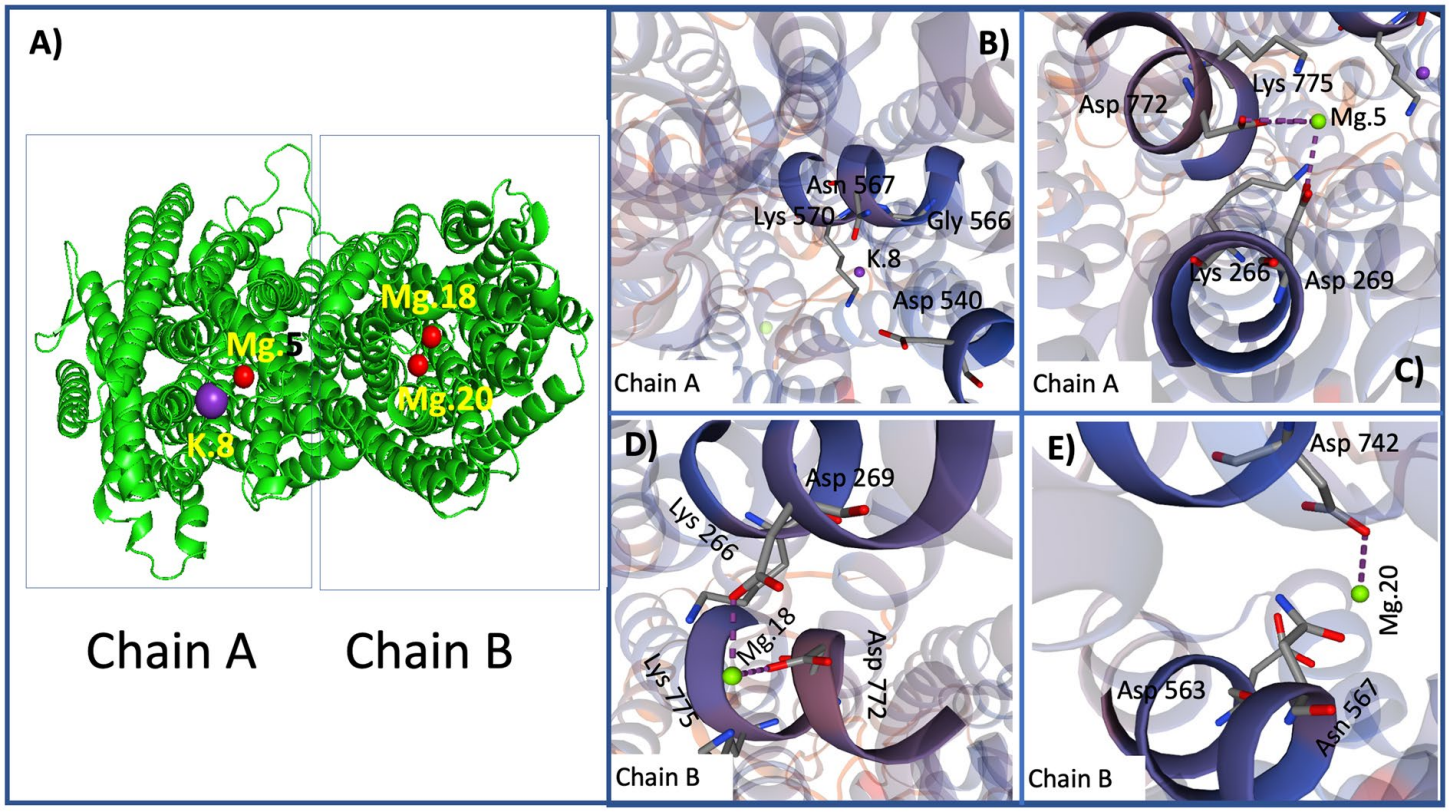
(**A**) Modelling of the three-dimensional structure corresponding to the protein sequence of the isoenzyme PdVP2 of the H^+^-PPase of *P. dicentrarchi*. The homodimeric type oligomeric modelling obtained from the quaternary structure is included, indicating the existence of two monomeric chains A and B (circles). The model predicts one ligand for K^+^ and three ligands for Mg^+2^ (**B**–**E**). K.8 has four coordination residues, and no protein–ligand interactions (PLIPs) were detected (**B**). Mg. 5 presents four coordination residues and three PLIPs in the A chain forming metal complexes (**C**). Mg.18 has four coordination residues and two PLIPs in the B chain protein forming metal complexes (**D**). Mg. 20 has three coordination residues and one PLIP in the B chain forming metal complexes (**E**). Templates were obtained by an automated comparative protein modelling server of the Swiss Model (URL: https://swissmodel.expasy.org/).

For construction of the Swiss-Model models, the GMQE (Global Model Quality Estimation) estimate was prioritized for the quaternary structure^74^. Thus, applying this estimator for PdVP1, a score of 0.78 was obtained, which represents good reliability (values higher than 0.7 are usually considered reliable), while for PdVP2, the estimated score was 0.66, which can be considered within the limit of reliability. For the QMEAN estimator, which provides global absolute quality estimates for the entire structure (Studer et al., 2020), the estimated score for the PdVP1 was -3.10, indicating good quality (scores around 0 indicate good agreement between model structure and similarly sized experimental structures, while scores of -4.0 or less indicate low-quality models). However, the QMEAN estimator gives the PdVP2 a value of -4.60, indicating a low-quality model. For both PdVP1 and PdVP2, the protein structure modelling of H^+^-PPases of *P. dicentrarchi* by the Swiss-Model predicts a quaternary structure of the oligomeric type sequence consisting of two homodimeric type chains (A and B) (matching prediction) (Figs. 2, 3A).

The model constructed for the PdVP1 protein establishes the existence of 3 × Mg^2+^ ligands (Fig. 2B–D). Mg.5 binds to amino acids K.219, D.222, D.699 and K.702 of chain A, two PLIPs and two interactions are generated forming metal complexes with the amino acids D.222 and D.699 of chain A (Fig. 2B). The Mg.6 binds to amino acids K.219, D.252 and D.471, forming two protein–ligand interactions (PLIPs) and metal complexes with the amino acids D.252 and D.471 of chain A (Fig. 2C). The Mg.19 binds to amino acids K.219, D.222, D.699 and K.702 of chain B generating 2 PLIPs and metal complexes with amino acids D.222 and D.699 of the chain B (Fig. 2D).

The PdVP2 forms ligands with 1 × K^+^ and 3 × Mg2^+^ (Fig. 3B–E). The potassium ion K.8 binds to amino acids D.540, G.566, N.567, K.570 of the A chain and no PLIPs were detected (Fig. 3B). The Mg.5 binds to amino acids K.266, D.269, D.772 and K.775 of chain A, generating 3 PLIPs with chain A and metal complexes with amino acids D.269, A: D. 772, and A: K.775 (Fig. 3C). The Mg.18 forms ligands with amino acids K.266, D.269, D.772 and K.775 of chain A, 2 PLIPs and metal complexes with amino acids D.269 and B: D.772 (Fig. 3D). Finally, the Mg.20 forms ligands with amino acids D.563, N.567 and D.742 of chain B and a PLIP and metal complexes with amino acid D.742 of chain B (Fig. 3E).

When we carried out the alignments to establish the phylogenetic relationships between species that possess H^+^-PPases of types VP1 and VP2 (Fig. five), we observed a single cysteine in each: C.601 in PdVP1 and C.669 in PdVP2. The cysteine is highly conserved and could possibly form disulfide bonds between monomers and generate the dimers of these enzymes (see Supplementary material, Fig. 1).

### Biochemical analysis and subcellular localization of P. dicentrarchi H^+^-PPases

**Figure 4:**
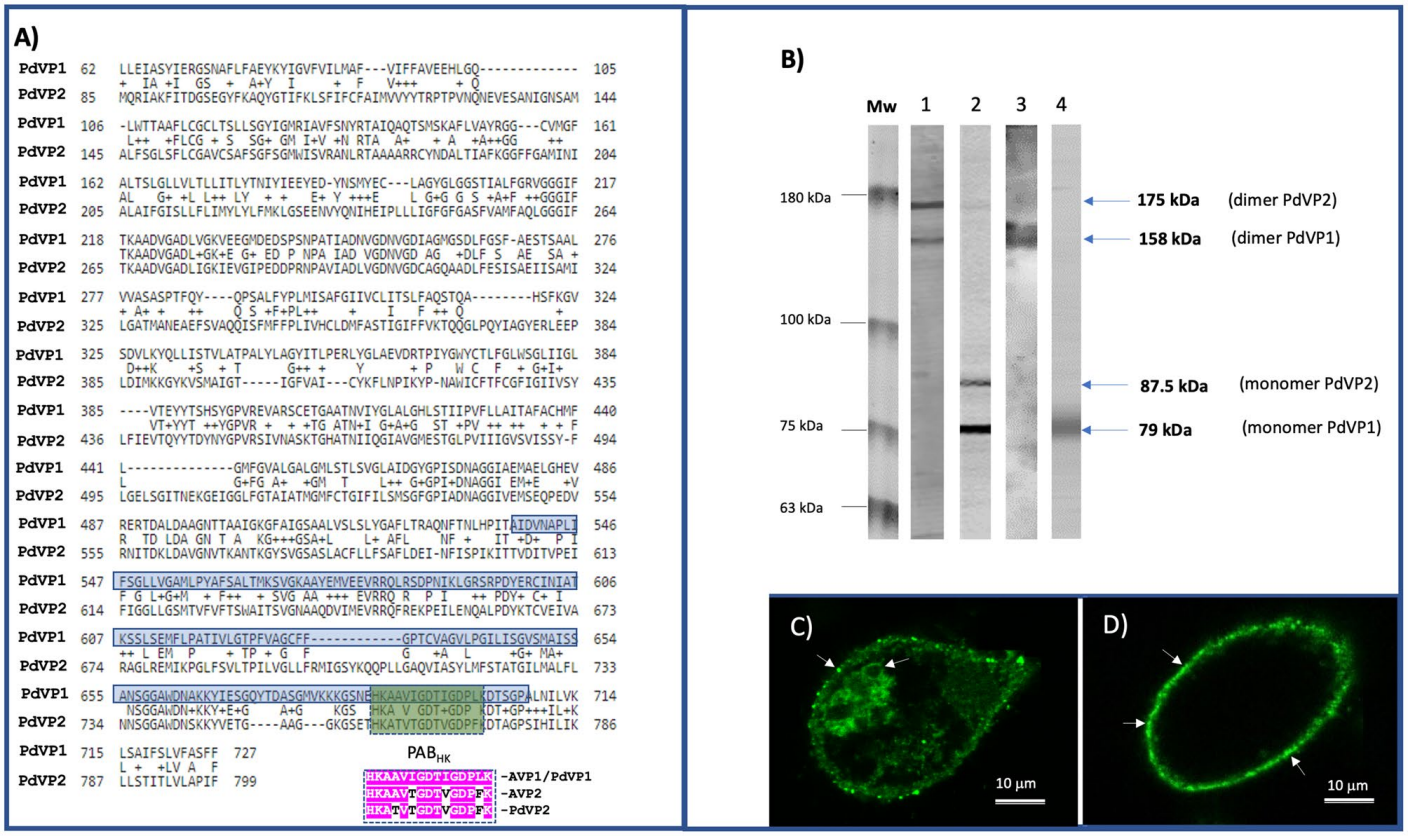
(**A**) Alignments of the aa sequences of the PdVP1 and PdVP2 isoenzymes of H^+^-PPases of *P. dicentrarchi* using the Blastp program (https://blast.ncbi.nlm.nih.gov/Blast.cgi). The blue shaded sequence corresponds to the fragment of the aa sequence of the PdVP1 isoenzyme expressed as a recombinant protein in the yeast *Klyuveromyces lactis* (rPdVP1), and the green shaded sequence corresponds to the HKAAVIGDTIGDPLK (HK) peptide of the isoenzyme PdVP1. The alignments of the PAB_HK_ motif in the V-H^+^-PPases AVP1 and AVP2 of *Arabidopsis thaliana* and the V-H^+^-PPases PdVP1 and PdVP2 of *P. dicentrarchi*, shown in pink shading, represent the common regions. (**B**) Western blot analysis of components of a *P. dicentrarchi* ciliate lysate (CL) separated by SDS-PAGE under non-reducing (-DTT; lanes 1 and 3) and reducing (+ DTT; lanes 2 and 4) conditions in the presence of polyclonal mouse antibodies α-rPdVP1 (lanes 1 and 2) and α-KLH-HK (lanes 3 and 4). Mw: molecular weight markers in kDa. (**C**,**D**) Immunolocalization of H^+^-PPases of *P. dicentrarchi* by immunofluorescence by use of polyclonal antibodies α-rPdVP1(**C**) and α-KLH-HK (**D**) with arrows indicating labelling sites.

We initially performed an alignment with the Blastp program to determine the degree of identity and similarity of the two sequences corresponding to the PdVP1 and PdVP2 of *P. dicentarchi* (Fig. 4A). Both sequences were found to have an identity of 33.5% and a similarity of 51.2%, with a gap of 16.2% (Fig. 4A). The same figure also shows the alignments obtained between the sequence belonging to the HKAAVIGDTIGDPLK motif (PAB_HK_ motif) characteristic of the V-H^+^-PPase type I (AVP1) of *Arabidopsis thaliana* and the sequences of the same motif present in the V-H^+^-PPasses PdVP1 and PdVP2 of *P. dicentrarchi* and AVP2 of *A. thaliana*.

The alignments between the PAB_HK_ motif sequences of AVP1 and PdVP1 indicate that they share the same sequence; however, comparison of the same sequence with AVP2 revealed three changes, and comparison of this motif in AVP2 and PdVP2 revealed only one change (Fig. 4A). Biochemical characterization and cellular localization of the H^+^-PPases of *P. dicentrarchi* was carried out by western-blot (WB) and immunofluorescence, respectively (Fig. 4B–D). For the WB assay, mouse-produced polyclonal antibodies were used against a fragment of the PdVP1 protein that was cloned and expressed in yeast (α-rPdVP1) and against a common peptide with the AVP1 motif PAB_HK_ conjugated to KLH (α-KLH-HK). In order to verify the existence of a potential oligomeric state in these enzymes, the proteins from a *P. dicentrarchi* CL were separated by SDS-PAGE under reducing (+ DTT) and non-reducing (-DTT) conditions and tested against α-rPdVP1 and α-KLH-HK antibodies by WB. The results obtained indicate that the α-rPdVP1 antibody recognizes two bands of 178 and 158 kDa under non-reducing conditions (Fig. 4B, lane 1), while both antibodies recognize two bands of 87 and 79 kDa under reducing conditions (Fig. 4B, lane 2). When α-KLH-HK antibodies were included in the WB, a single 158 kDa band was observed under non-reducing conditions (Fig. 4B, lane 3) and a 79 kDa band was observed under reducing conditions (Fig. 4B, lane 4). In addition, we also predicted the location of the H^+^-PPases of *P. dicentarchi* by using the LocTree 3 program, which enabled us to conduct a bioinformatic prediction of subcellular localization. The findings indicated that at least some of the peptide sequence of PdVP1 is embedded in the hydrophobic region of the membrane vacuole and that in PdVP2 some of the sequence is in the Golgi apparatus membrane (Supplementary material, Fig. 2). By contrast, immunofluorescence assays showed that α-KLH-HK antibodies generate specific immunostaining below the plasma membrane where the alveolar sacs are located (Fig. 4D), while the α-rPdVP1 antibody generates immunostaining both in the alveolar sacs and in the cytoplasmic vacuoles (Fig. 4C).

### Phylogenetic analysis

**Figure 5:**
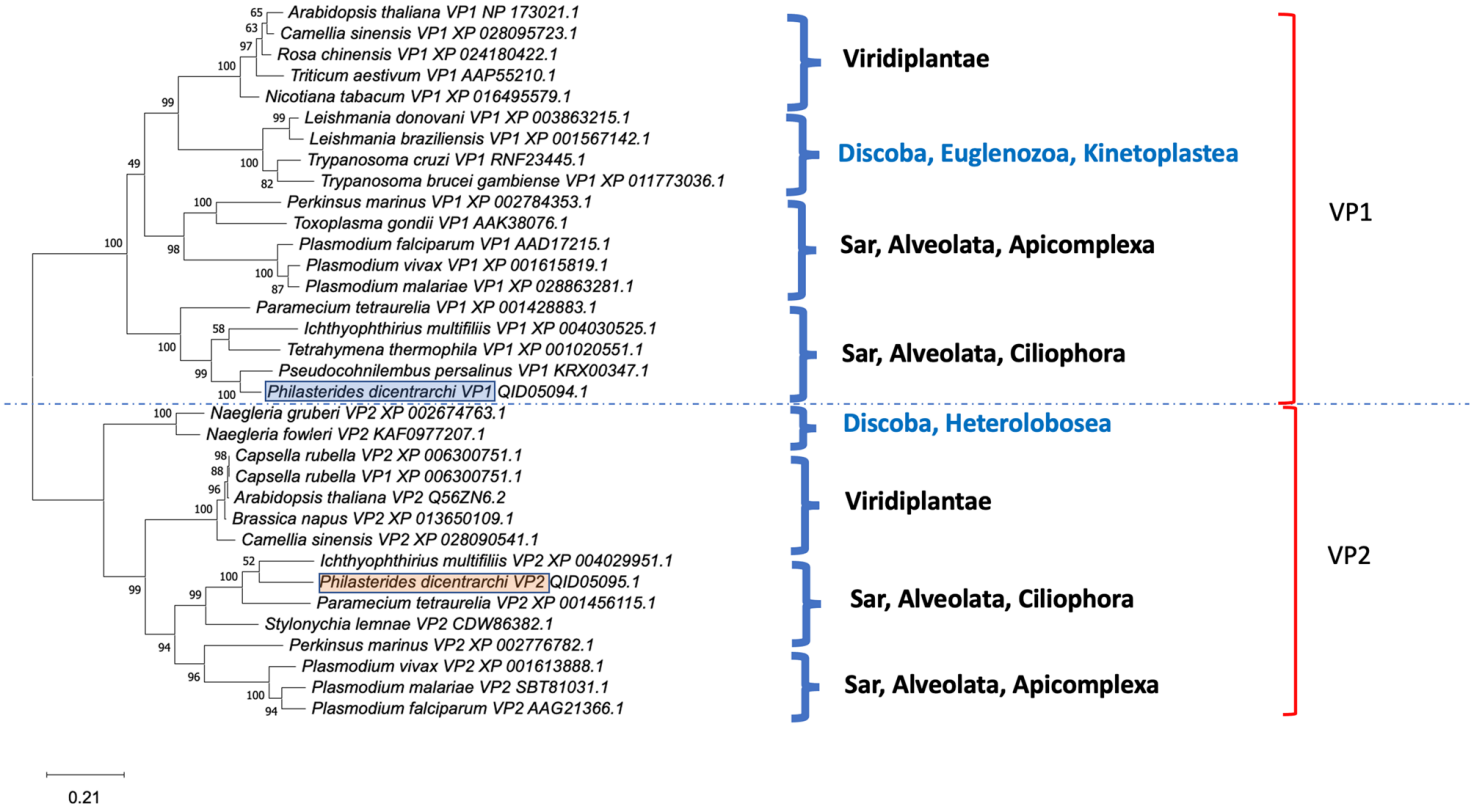
Phylogenetic analysis of aa sequences of *P. dicentrarchi* isoenzymes PdVP1 and PdVP2 inferred by Maximum likelihood method and JTT matrix-based model. The GenBank access number and the type of H^+^-PPase (vacuolar pyrophosphatase type 1 and 2, VP1-VP2) is included after the name of the species. The tree with the highest log likelihood (− 21,198.33) is shown. The percentage of replicate trees in which the associated taxa clustered together in the bootstrap test (500 replicates) is shown beside the branches. Initial trees for the heuristic search were obtained automatically by applying Neighbour-Join and BioNJ algorithms to a matrix of pairwise distances estimated using a JTT model and then selecting the topology with the highest log likelihood value. The tree shown is drawn to scale, with branch lengths measured in the number of substitutions per site. All positions containing gaps and missing data were eliminated using the TrimAl program. This analysis involved 34 amino acid sequences and a total of 621 positions in the final data set. Evolutionary analyses were conducted with MEGA X software.

We used the Maximum Likelihood method (ML) to conduct the phylogenetic analysis. To analyze the degree of identity of the *P. dicentrarchi* PdVP1 and PdVP2 sequences with sequences deposited in the NCBI database belonging to plant and protist species, we performed a basic local alignment using the BLASTp tool. The results presented in Fig. 5 indicate that, like *P. dicentrarchi*, other ciliates possibly also possess two types of H^+^-PPases. On the one hand, PdVP1 has the highest identity with a hypothetical protein PPERSA_10846 from the scuticociliate *Pseudocohnilembus persalinus* and, to a lesser degree, with other hypothetical proteins of the ciliate *Paramecium tetraurelia*, with a putative inorganic pyrophosphatase of *Ichthyophthirius multifiliis* and with an inorganic pyrophosphatase from *Tetrahymena thermophila* (Fig. 5). On the other hand, the PdVP2 of *P. dicentrarchi* has the highest identity with a vacuolar-type H^+^-PPase putative *of I. multifiliis*, with a hypothetical protein of *P. tetraurelia* and with a pyrophosphatase-energized 3-like membrane proton pump of the ciliate *Stylonychia lemnae* (Fig. 5).

Although in *P. dicentrarchi* both PdVP1 and PdVP2 are, from a phylogenetic point of view, closely related to other H^+^-PPases of ciliate species, PdVP1 is evolutionarily close to the H^+^-PPases of plants and kinetoplastid protists, while PdVP2 is phylogenetically more closely related to H^+^-PPases of other protists such as amoebae and apicomplexans (Fig. 5).

### Effects of various stimuli, antiprotozoal drugs, and infection on transcription of the H^+^-PPases in *P. dicentrarchi*

**Figure 6:**
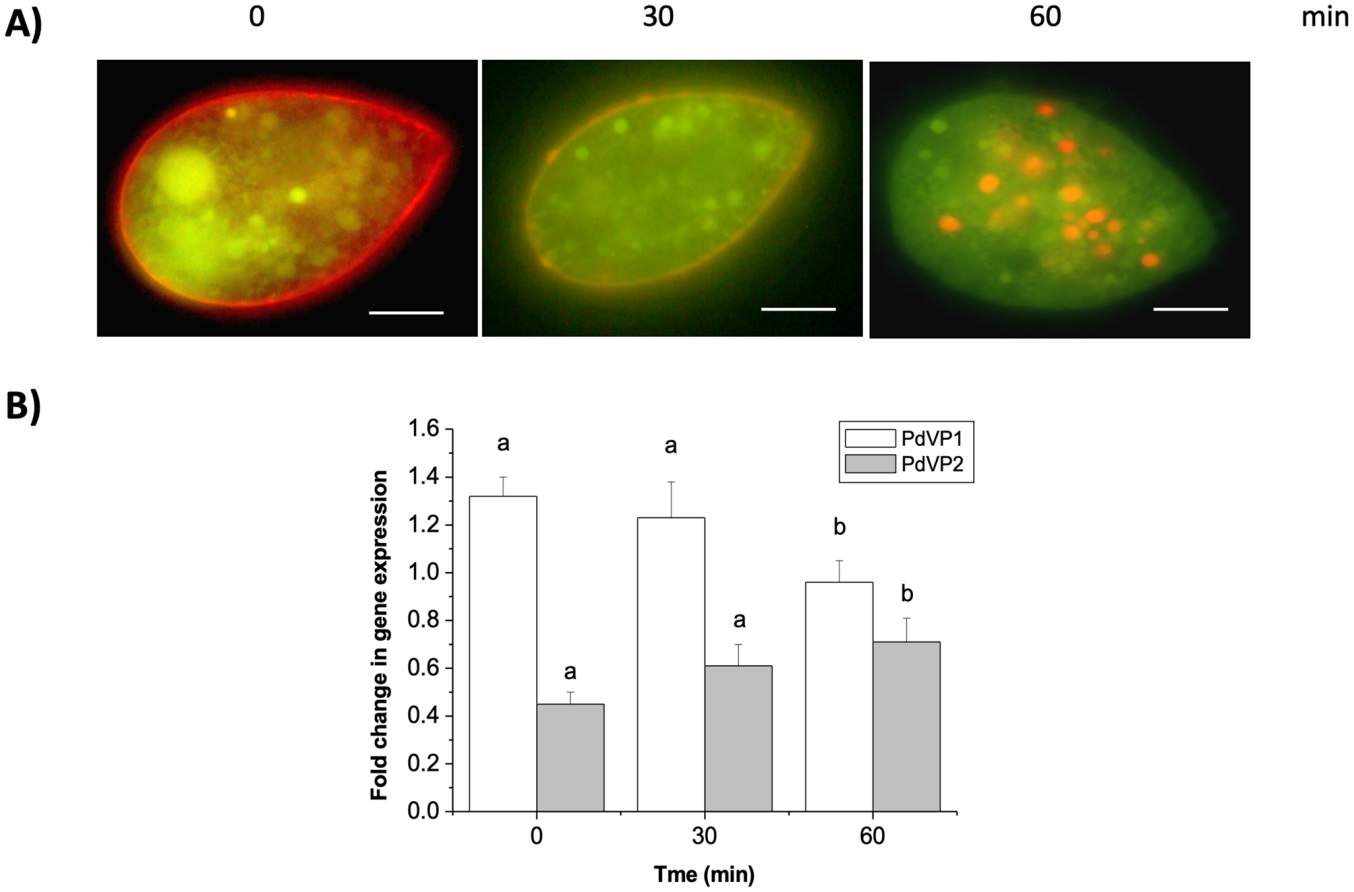
(**A**) Changes in the acidification kinetics of the alveolar sacs and cytoplasmic vacuoles after the addition of Tris-PPi (1 mM) and MgSO_4_ (1.3 mM), and after 30 and 60 min (detected by fluorescence microscopy). An increase in acidification was detected by the change in fluorescence emission in the acridine orange stain from green (not acidified) to red (acidified). (**B**) Quantification of the expression of the H^+^-PPases (PdVP1 and PdVP2) obtained under the same conditions as (**A**). The bars express the mean values ± the standard error (SE) (n = 5) in the fold change in gene expression. The same and different letters indicate respectively the absence or presence of statistically significant differences (*P* < 0.05) relative to the control (time 0). Scale bar = 10 μm.

In one experiment, the effect of the addition of PPi and Mg^2+^ on intracellular acidification (Fig. 6A) of trophonts and on the expression of the H^+^-PPases of *P. dicentrarchi* was evaluated at different incubation times (0, 30 and 60 min) (Fig. 6B). After addition of PPi and Mg^2+^, very rapid and intense acidification was immediately observed at the periphery of the ciliate at the level of the alveolar sacs (Fig. 6A). Acidification of the alveolar sacs gradually decreased until disappearing completely, after 60 min (Fig. 6A).

By contrast, acidification of the intracytoplasmic vacuoles increased over time, reaching maximal intensity 60 min after the stimulus (Fig. 6A). Evolution of the transcription of the isoenzymes of H^+^-PPases also followed the same trend as the acidification kinetics (Fig. 6B). Thus, the transcription of isoenzyme PdVP1 decreased over time, while the transcription of isoenzyme PdVP2 increased over time (Fig. 6B).

**Figure 7:**
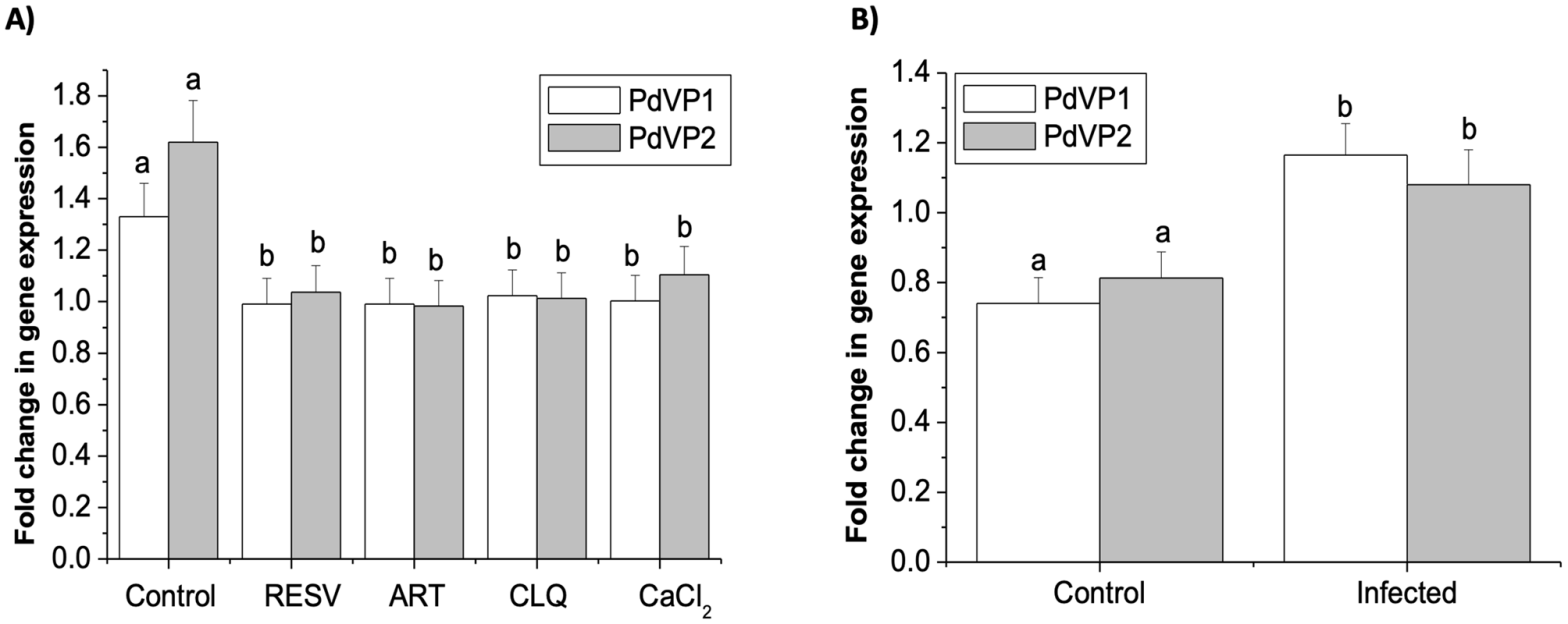
Analysis of the expression of the PdVP1 and PdVP2 isoenzymes of the *P. dicentrachi* H^+^-PPases. (**A**) PdVP1 and PdVP2 transcription in trophonts incubated for 2 h with 100 µM of the antiprotozoals resveratrol (RESV), artemisinin (ART) and chloroquine (CLQ) and 100 mM of CaCl_2_. (**B**) Transcription levels of PdVP1 and PdVP2 isoenzymes in trophonts obtained 2 h after intraperitoneal infection of turbot (infected) relative to the same trophonts that were maintained in in vitro culture (control). The results presented in the graph correspond to the mean values ± the standard error (SE; n = 5) in the fold change in gene expression. Statistically significant difference between groups are indicated by different letters (*P* < 0.05).

The expression of both PdVP1 and PdVP2 isoenzymes also decreased significantly after the addition of antiparasitic molecules such as the polyphenol resveratrol (RESV), the antimalarial drugs artemisinin (ART) and chloroquine (CLQ), and also after the addition of CaCl_2_ (Fig. 7A). By contrast, when the ciliate was in the endoparasitic phase in the turbot, the expression of both isoenzymes increased significantly (Fig. 7B).

## Discussion

In plants and other organisms, H^+^-PPases are pumps that drive the generation of a H + -gradient across the membranes of vacuoles, Golgi apparatus and endosomes by utilizing PPi as a substrate^31,34,75^. In previous studies, we have shown that the amphizoic scuticociliate *P. dicentrarchi*, which parasitizes farmed turbot, has H^+^-PPases located in the membrane of vacuoles and alveolar sacs and that these share characteristics with other H^+^-PPases of protists and plants^22,47^. Most plants have two types of H^+^-PPases^13^: type I requires K^+^ and is located in the vacuole membranes, while type II does not require K^+^ and is located in the membrane of the Golgi apparatus and related membranes^34^. Apart from plants, two types of H^+^-PPases have also been described in various parasitic protists^47,76,77^. The description of the *P. dicentrarchi* H^+^-PPase was initially based on a cDNA sequence obtained by RACE-PCR, which we assumed included the complete sequence of this enzyme, containing 587 amino acids and with a molecular mass of 61.7 kDa and an isoelectric point (IP) of 5.0^22^. In this study, we confirmed that the mRNA encoding the complete H^+^-PPase type 1 has an open reading frame (ORF) of 2241 bp, compared to the 1761 bp originally described, which encodes a protein of 746 aa, weighing molecular estimate of 78,947.27 Da and a theoretical IP of 5.09. These data indicate that the *P. dicentrarchi* H^+^-PPase that we initially characterized was a partial sequence of PdVP1^22^. The biochemical parameters of sequence size, molecular weight and IP of *P. dicentrarchi* PdVP1 are similar to those of *Arabidopsis thaliana* V-H^+^-PPase type I (AVP1), which has a sequence of 770 amino acids and a molecular weight of 80.8 kDa monomer^78^. Several motifs characteristic of plant vacuolar H + -PPases were also found in the PdVP1 isoenzyme of *P. dicentrarchi*, which contains several sequence motifs of the prototypical type I V–H^+^-PPase from *A. thaliana* vacuolar pyrophosphatase type I (AVP1)^22^. Thus, the IADNVGDNVGD domain present in both AVP1 and PdVP1 is a highly conserved domain in the pyrophosphate-energized vacuolar membrane proton pump 1 of plants^3^. PdVP1 also possesses another highly conserved domain HKAAVIGDTIGDPLK characteristic of AVP1 from *A. thaliana*^79^ and from the type I vacuolar H^+^-translocating pyrophosphatase (TgVP1) from the apicomplexan *Toxoplasma gondii*^80^.

The H^+^-PPase type 2 (PdVP2) of *P. dicentrarchi* has a mRNA sequence of 2433 bp that encodes a protein of 810 aa, with an estimated molecular weight of 87,596.32 Da and a theoretical IP of 5.36. In the *A. thaliana* AVP2 gene, it generates an ORF of 2403 bp encoding an 800 amino acid (81 KDa) polypeptide^13^, biochemical parameters like those presented by *P. dicentrarchi* PdVP2. The isoenzyme PdVP2 from *P. dicentrarchi* also has a highly conserved motif, IPEDDPRNPAVIADLVGDNVGDCA, in common with AVP2 from *A. thaliana*^13^ and with H^+^-PPase type II (PfVP2) from apicomplexan *P. falciparum*^76^. Another characteristic motif of AVP2, HKAAVTGGDTVGDPFK ^13,29^, is also present in the *P. dicentrarchi* PdVP2 showing only one change in the aa of the fourth residue A (AVP2), which is replaced by T (PdVP2). From a topological point of view, the predictions indicate a high degree of similarity between the V-PPases of plants and parasites containing between 16–17 TMRs associated with helix-forming membranes, six of which form internal rings that participate in the translocation of protons^81–83^. Analysis of the transmembrane topology of *P. dicentrarchi* PdVP1 by using the Phobius program predicted the presence of 13 TMRs associated with the vacuole membrane also containing six internal rings, which according to some authors participate in the pathway proton translocation^83^. This prediction of *P. dicentrarchi* PdVP1 topology is consistent with the number of TMRs described for *A. thaliana* AVP1^78^. On the other hand, the number of *P. dicentrarchi* PdVP2 TMRs (16) is one less than the number of TMRs (17) that the *A. thaliana* AVP2 possesses^34^. Two types of H^+^-PPases have also been found in some protists; e.g. in the kinetoplastid *Trypanosoma brucei* the type I H^+^-PPase has 15 TMRs associated with the membrane of vesicular-like acidocalcisomes^77,84,85^. The malaria parasite *Plasmodium falciparum* also has two types of H^+^-PPases (PfVP1 and PFVP2) that have 15 TMRs in their aa sequences^76^. The PfVP1 has a 51% identity (60% similarity) with the prototypical type I plant AVP1; however, PfVP1 shares only 30–40% identity (49–50% similarity) with AVP2; on the contrary, PfVP2 shares 50% identity (59% similarity) to AVP2 but only 39% identity (49% similarity) to PfVP1^76^ (see Supplementary material, Fig. 3). The PdVP1/ PdVP2 of *P. dicentrarchi* shares 33.5% sequence identity and 51.2% similarity, values like those presented between the AVP1/AVP2, which shares 36% sequence identity and 51% similarity^78^. Comparison of the degree of identity / similarity between the aa sequences of PdVP1/AVP1 sharing 52% identity and 67% similarity, and PdVP2/AVP2 sharing 55% identity and 74% similarity.

The substrate necessary for H^+^-PPase activation is an MgPPi complex^86–89^, with Mg^2+^ acting as an allosteric activator of the enzyme^90^. In the present study, after prediction of the protein structures of PdVP1 and PdVP2 in *P. dicentrarchi*, we detected three Mg^2+^ binding sites in the chains of these enzymes and an additional K^+^ binding site in PdVP2. Although bioinformatic modelling does not predict any K^+^ binding site in PdVP1, a site in the protein sequence determines strict K^+^-dependence^22^. As in the other V-PPases, the binding sites for Mg^2+^ of PdVP1 and PdVP2 are found in the hydrolytic centre, which comprises several aa residues from the larger helices of the inner ring TMRs that facilitate the translocation of a proton from the cytosolic domain and the release of the proton to the vacuolar lumen^83^.

Vacuolar-PPases (V-PPases; EC 3.6.1.1) belong to the family of membrane integral hydrophobic PPases, which are homodimeric proteins made up of monomers of molecular weight 70–81 kDa^3,12,82,91–94^. Bioinformatic predictions about the protein structure homology-modelling derived from the aa sequences of *P. dicentrarchi* PdVP1 and PdVP2 indicate that these isoenzymes have an oligomeric structure of homodimeric type consisting of two monomers. These results were confirmed by WB analysis with α-rPdVP1 antibodies and an α-KLH-HK antibody (similar to anti-PAB_HK_) containing a highly conserved motif in V-H^+^-PPases^12,47^. Under non-reducing conditions, the α-KLH-HK antibody recognizes a 158 kD band, while under reducing conditions this antibody recognizes two 78 kDa bands, indicating that the native protein of PdVP1 is an oligomer composed of 2 monomers (homodimer). The native protein of PdVP2 is also a 175 kD homodimer and consists of two identical 85.7 kDa polypeptide chains, somewhat larger than the *A. thaliana* AVP2 (81 kDa)^13^. Cysteine residues corresponding to C.601 of PdVP1 and C.669 of PdVP2 are highly conserved in VP1 and 2 H^+^-PPases, which may indicate their potential participation in the formation of intermolecular disulfide cross-linking to generate oligomers, as occurs in bacteria and in other organisms^7,95^.

The bioinformatic prediction of the subcellular localization of *P. dicentrarchi* PdVP1 in the membrane of intracytoplasmic vacuoles also coincides with *A. thaliana* AVP1, which is also located in the membrane of the vacuole and endosomes^11,22,32^, while the prediction of PdVP2 indicates a location on the Golgi apparatus membrane, like the AVP2 of *A. thaliana*^29,34^. We have previously shown through immunofluorescence studies that *P. dicentrarchi* trophonts possess vacuolar and alveolar-membrane-located H^+^-PPases that are probably related to PdVP1 and PdVP2 respectively^47^. However, we also proposed that the *P. dicentrarchi* H^+^-PPase possess two isoforms that share the PAB_HK_ motif, with isoform 2 being attributed a location in the alveolar sacs^47^; however, this possibility has been ruled out in the present study. Alveoli consist of single membrane flattened sacs probably derived from the endomembrane system^96^ which are typical of the Alveolates, a group of unicellular eukaryotes what includes apicomplexan parasites, dinoflagellate algae and ciliates^97^. In the parasitic ciliate fish *Ichthtyophthirius multifiliis*, both alveolar sacs and the thick membrane cisternae of the cell cortex (Golgian-like cisternae) display pyrophosphatase activity, which indicates a close relationship between the alveolar sacs and the Golgi complex^98^. In the present study, the immunofluorescence results indicate that the antibodies directed against the PABHK motif, which characterizes the isoenzyme PdVP1 and which is different in the PdVP2, produce specific immunostaining on the alveolar sacs, while the antibodies generated against the rPdVP1, which shares common sites with PdVP2, produces a label with both the alveolar sacs and the cytoplasmic vacuoles, indicating that PdVP2 is probably located in the vacuoles. On the other hand, the immunofluorescence results contradict the bioinformatic predictions of subcellular location (see Supplementary material, Fig. 2), which clearly highlights the need to contrast and validate the results proposed by the predictive models with the results obtained in the experimental tests.

V-H^+^-PPase is a highly conserved and highly ubiquitous enzyme in eukaryotes, normally forming monophyletic groups, indicating that it is an ancient protein that emerged before the last universal common ancestor (LUCA) in life diversification^12,21^. From a phylogenetic point of view, it has been demonstrated that both PdVP1 and PdVP2 represent two paralogues of the *P. dicentrarchi* V-H^+^-PPase gene as well as the rice and wheat V-H^+^-PPase genes^99,100^. Two fragments of DNA with different sequences have also been identified in *P. tetraurelia* and *T. pyriformis*, indicating the existence of two paralogous H^+^-PPase genes in these ciliates^101^. In some protozoan and plant species the same genes encode membrane integral hydrophobic PPases; however, the presence of two different paralogous H^+^-PPase genes is relatively common^13,17,21^. These two types of isoenzymes also appear in various ciliate species, showing high similarities with both PdVP1 and PdVP2 from *P. dicentrarchi* and forming two monophyletic groups in which PdVP1 is more closely related to the V-H^+^-PPases of the plants and kinetoplastid protists, while PdVP2 is more closely related to V-H^+^-PPases of amoebas and apicomplexans.

In plants, V-H^+^-PPases can replace V-ATPases under energy stress to maintain the acidity of the vacuole and the intracellular pH, both of which are essential for cell viability^3,102,103^. In *P. dicentrarchi*, both intracellular vacuoles and alveolar sacs have H^+^-PPases associated with their membranes. After the addition of PPi and Mg^2+^, the PPases cause acidification of these cellular compartments, behaving like the acidocalcisomes of other protists^56^. The cell acidification kinetics in *P. dicentrarchi* start rapidly in alveolar sacs and acidification of intracellular vacuoles then occurs. The acidification kinetics also occur at the level of expression of the PdVP1 and PdVP2 isoenzymes, initially increasing the transcription of PdVP1 and later the transcription of PdVP2. The presence of the membrane-associated isoenzyme PdVP1 of the subplasmalemal calcium stores and PdVP2 forming part of the membranes of intracellular vacuoles may indicate some similarity to acidocalcisomes-like structures^104–106^.

Due to the absence of V-H^+^-PPases in animals, these enzymes have been proposed as targets for drug development^107^. Previous studies have shown that antimalarial drugs such as chloroquine (CQ) and artemisinin (ART) exert an antiparasitic effect against *P. dicentrarchi* by altering the intracellular pH of the parasite mediated by H^+^-PPases^57^. The polyphenol resveratrol (RESV) can alter histone acetylation through the enzyme class III histone deacetylase (HDCAs) in mammals and in protozoal parasites such as *Trypanosoma cruzi*, thereby altering the control of gene expression^108,109^. In *P. dicentrarchi*, RESV also acts as an antiparasitic agent, further affecting the cellular detoxification of reactive oxygen species^110,111^. All the antiparasitic agents tested in this study showed some ability to inhibit transcription of the two *P. dicentrarchi* H^+^-PPases, PdVP1 and PdVP2. Ca^2+^ is a known inhibitor of the enzymatic activity of plant H^+^-PPases^11,112^. In the present study, Ca^2+^ also demonstrated a significant inhibitory effect on the transcription of both PdVP1 and PdVP2 from *P. dicentrarchi*. During infection, the transcription of *P. dicentrarchi* H^+^-PPases PdVP1 and PdVP2 increased significantly, as does that of the V-H^+^-PPases (PfVP1 and PfVP2) of the malaria parasite *P. falciparum*, which also increases the expression of these genes in all the erythrocytic stages of infection^113^. This increase in the expression of H^+^-PPases may be related to mechanisms of adaptation of the ciliate to the osmotic stress conditions to which it is subjected inside the host during infection, in a similar way as during saline stress on plants^114^. In parasitic protists, V-H^+^-PPases are also necessary to maintain parasite virulence and survive the intracellular and extracellular environments^115,116^.

We have confirmed that the scuticociliate parasite *P. dicentrarchi* has two paralogous genes that encode two isoenzymes -PdVP1 and PdVP2- belonging to the family of integral membrane pyrophosphatases, which have a marked structural similarity with the V-H^+^-PPases AVP1 and AVP2 of *A. thaliana* and with integral membrane pyrophosphatases of other parasitic protozoa. *P. dicentrarchi* H^+^-PPases are homodimeric proteins that are in the intracellular vacuoles and in the alveolar sacs, participating in the maintenance of the intracellular pH and presenting a function like that of the acidocalcisomes of the parasitic protozoa. Genetic expression of the PdVP1 and PdVP2 isoenzymes is inhibited by antiparasitic drugs and increases during the endoparasitic phase, indicating a possible role for these enzymes in maintaining virulence and in the ability of this ciliate to adapt to endoparasitism.

## Supplementary Information

Supplementary Information.

## Data Availability

The datasets generated and/or analysed during the current study are available from the corresponding author on reasonable request.
