# Supplementary material for: Molecular characterization and transcriptional regulation of two types of H+-pyrophosphatases in the scuticociliate parasite Philasterides dicentrarchi
Source: Sci Rep. 2021 Apr 19;11:8519. doi: 10.1038/s41598-021-88102-0 (PMC8055999; doi:10.1038/s41598-021-88102-0)
Supplement: Supplementary file 1 — Supplementary Information. [file 41598_2021_88102_MOESM1_ESM.pptx]

## Slide 1
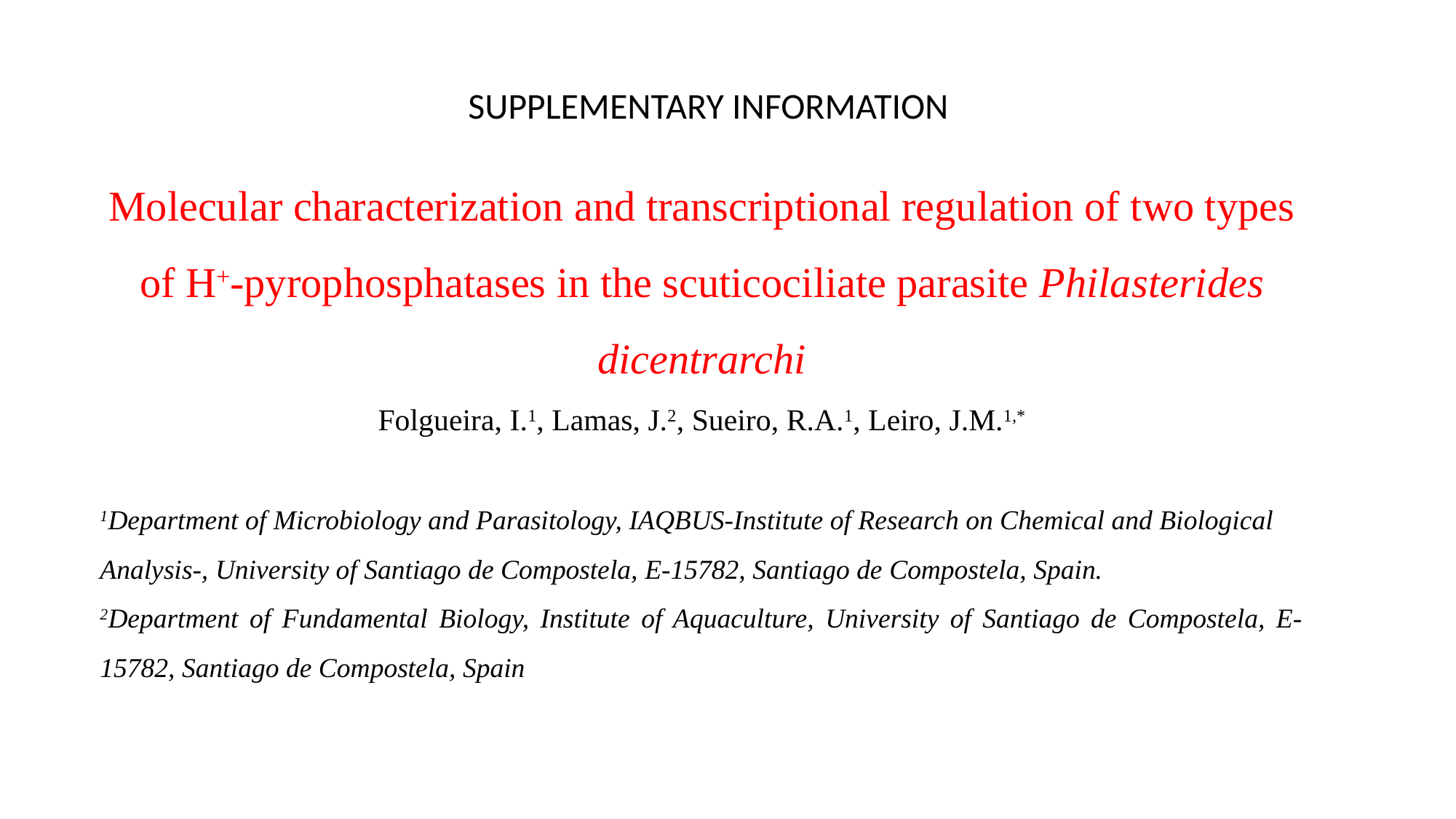

SUPPLEMENTARY INFORMATION
Molecular characterization and transcriptional regulation of two types of H+-pyrophosphatases in the scuticociliate parasite Philasterides dicentrarchi
Folgueira, I.1, Lamas, J.2, Sueiro, R.A.1, Leiro, J.M.1,*
1Department of Microbiology and Parasitology, IAQBUS-Institute of Research on Chemical and Biological Analysis-, University of Santiago de Compostela, E-15782, Santiago de Compostela, Spain.
2Department of Fundamental Biology, Institute of Aquaculture, University of Santiago de Compostela, E-15782, Santiago de Compostela, Spain

## Slide 2
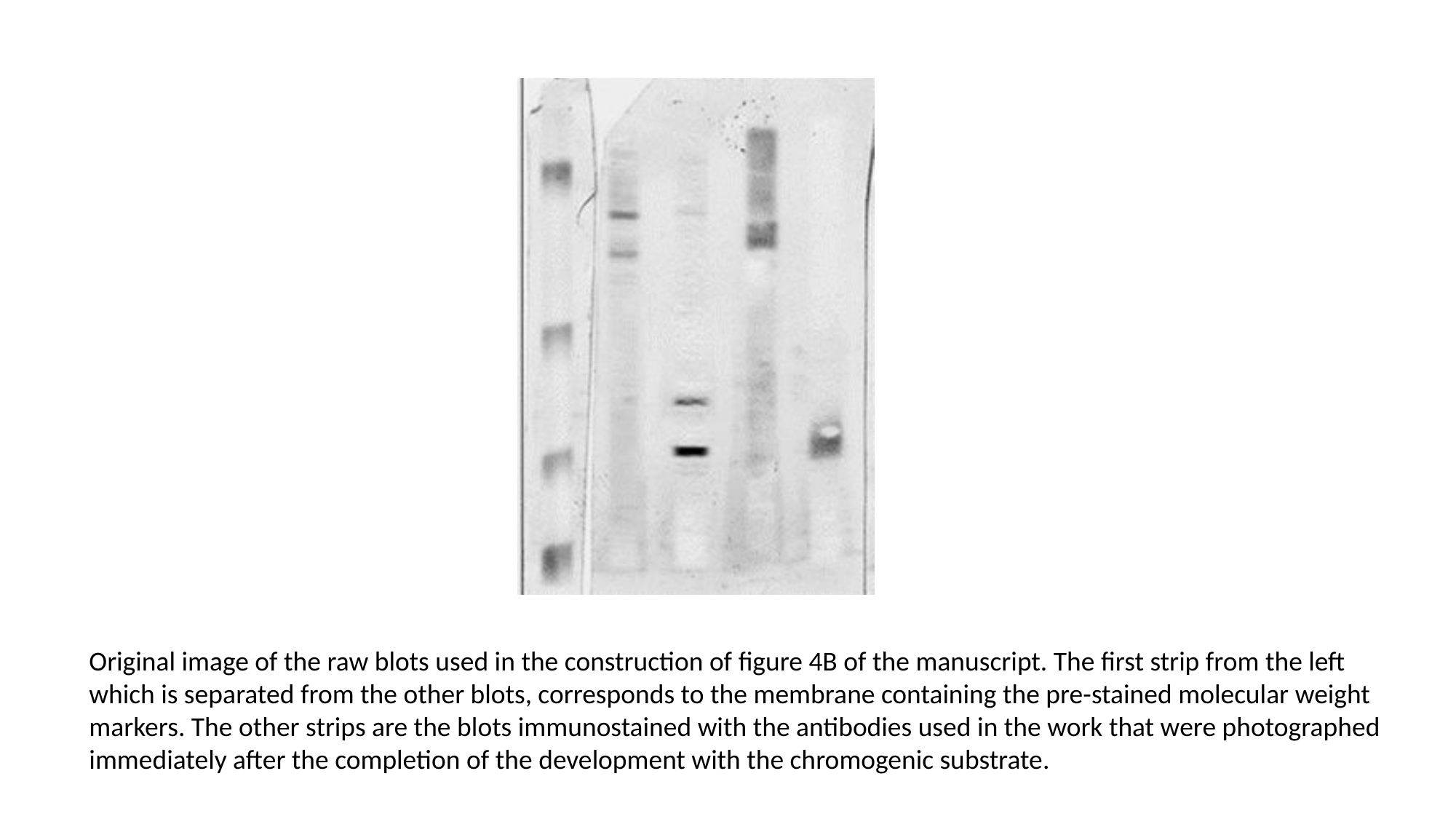

Original image of the raw blots used in the construction of figure 4B of the manuscript. The first strip from the left
which is separated from the other blots, corresponds to the membrane containing the pre-stained molecular weight markers. The other strips are the blots immunostained with the antibodies used in the work that were photographed immediately after the completion of the development with the chromogenic substrate.

## Slide 3
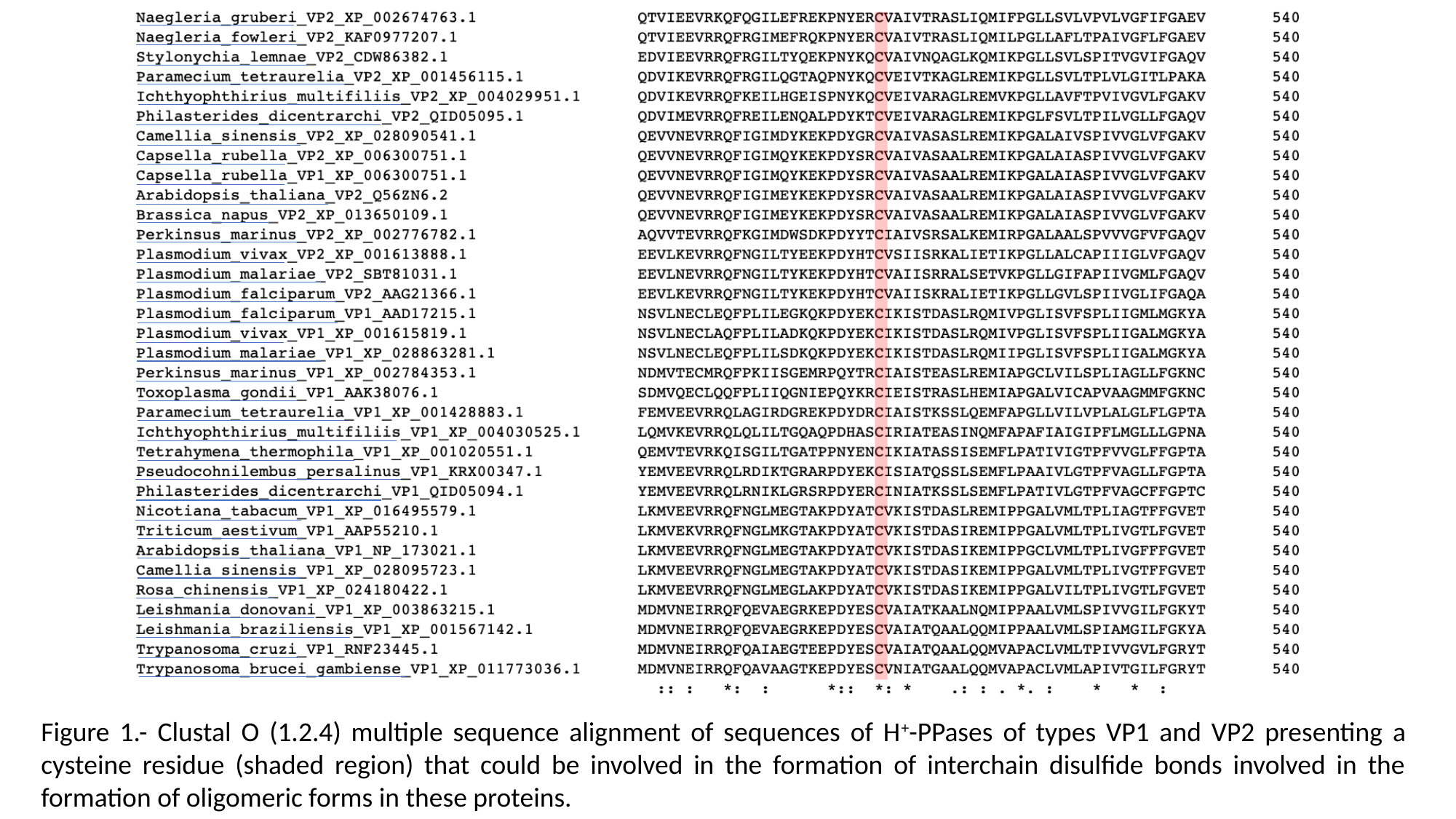

Figure 1.- Clustal O (1.2.4) multiple sequence alignment of sequences of H+-PPases of types VP1 and VP2 presenting a cysteine residue (shaded region) that could be involved in the formation of interchain disulfide bonds involved in the formation of oligomeric forms in these proteins.

## Slide 4
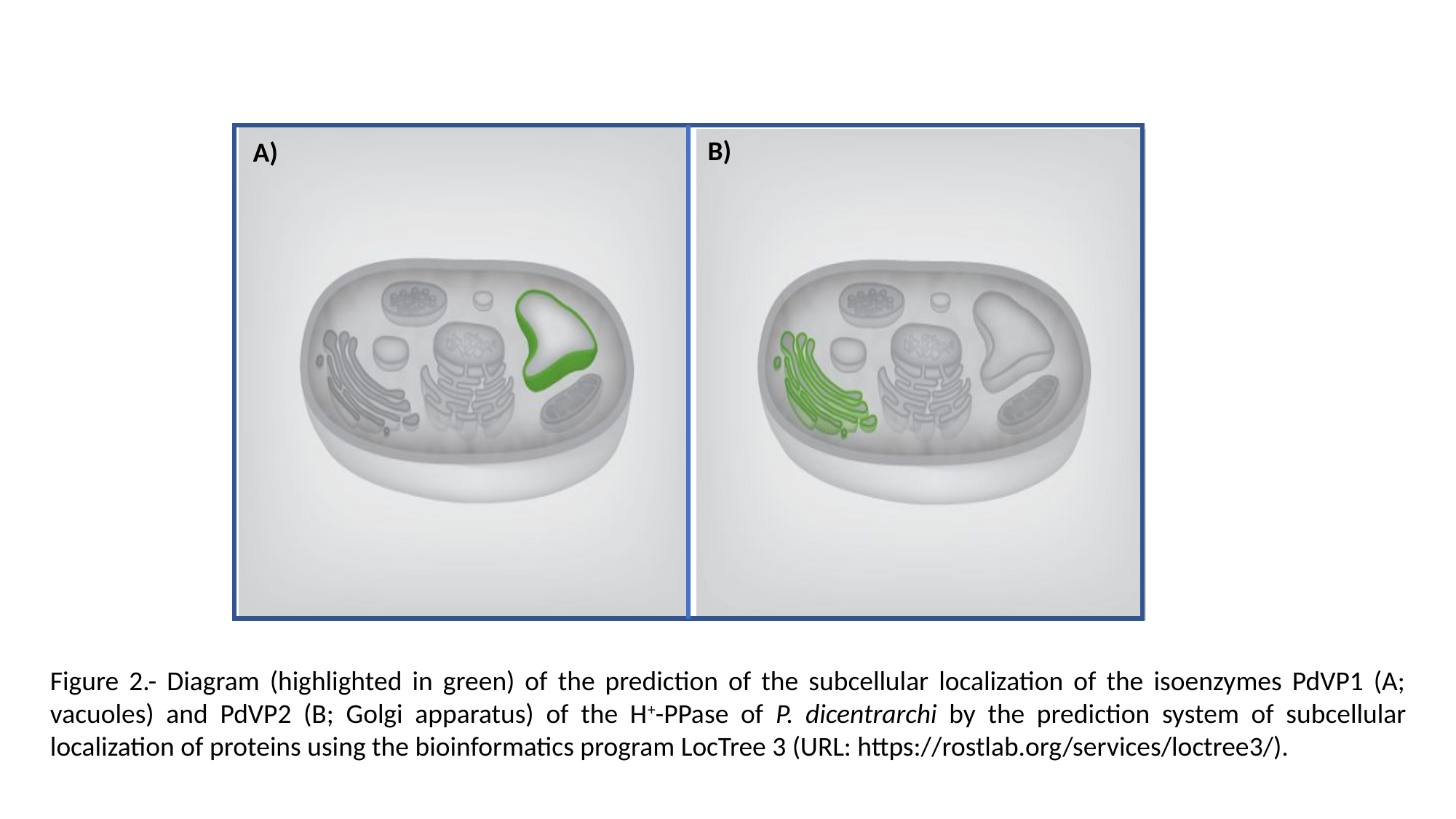

B)
A)
Figure 2.- Diagram (highlighted in green) of the prediction of the subcellular localization of the isoenzymes PdVP1 (A; vacuoles) and PdVP2 (B; Golgi apparatus) of the H+-PPase of P. dicentrarchi by the prediction system of subcellular localization of proteins using the bioinformatics program LocTree 3 (URL: https://rostlab.org/services/loctree3/).

## Slide 5
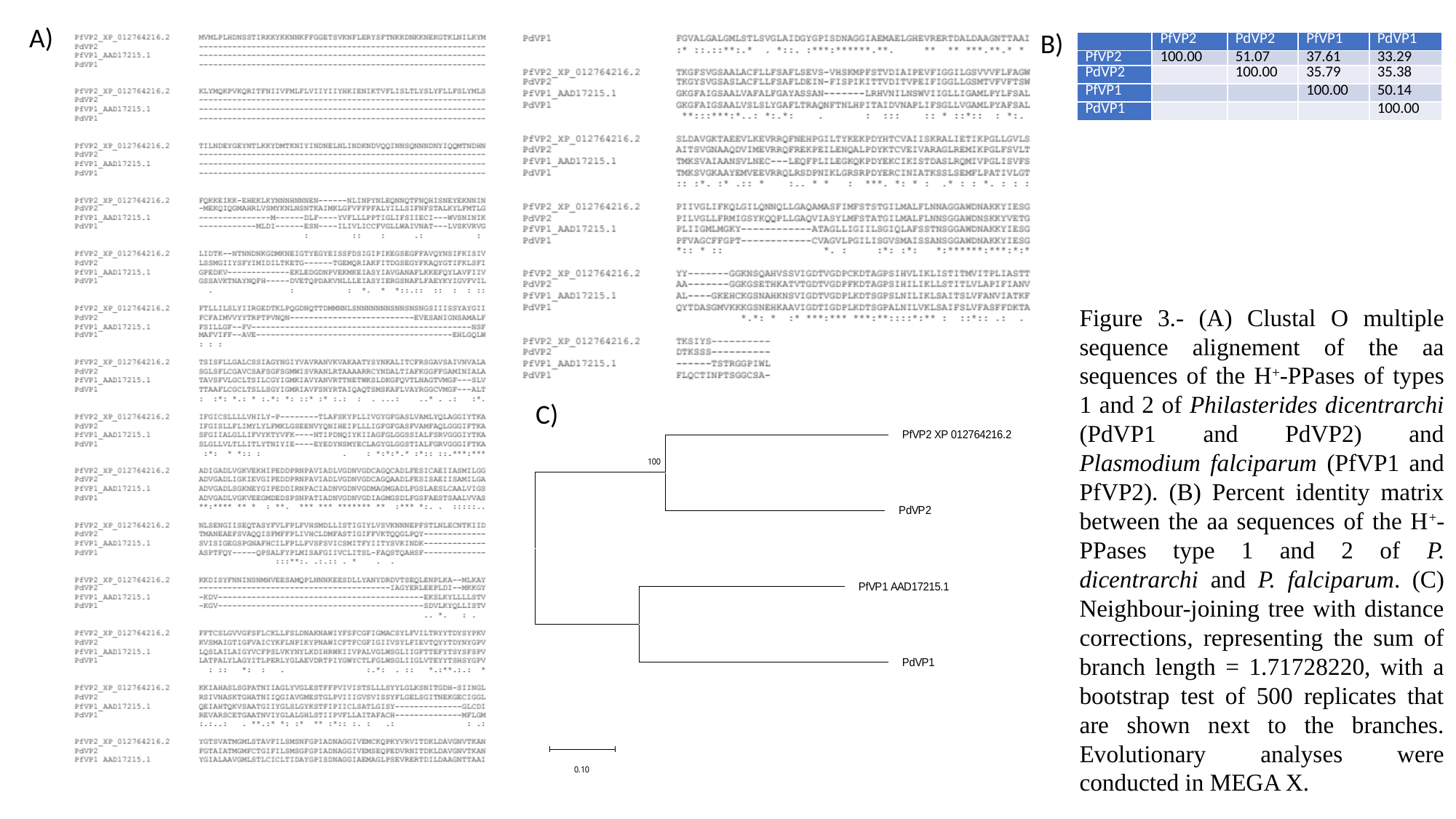

A)
B)
| | PfVP2 | PdVP2 | PfVP1 | PdVP1 |
| --- | --- | --- | --- | --- |
| PfVP2 | 100.00 | 51.07 | 37.61 | 33.29 |
| PdVP2 | | 100.00 | 35.79 | 35.38 |
| PfVP1 | | | 100.00 | 50.14 |
| PdVP1 | | | | 100.00 |
Figure 3.- (A) Clustal O multiple sequence alignement of the aa sequences of the H+-PPases of types 1 and 2 of Philasterides dicentrarchi (PdVP1 and PdVP2) and Plasmodium falciparum (PfVP1 and PfVP2). (B) Percent identity matrix between the aa sequences of the H+-PPases type 1 and 2 of P. dicentrarchi and P. falciparum. (C) Neighbour-joining tree with distance corrections, representing the sum of branch length = 1.71728220, with a bootstrap test of 500 replicates that are shown next to the branches. Evolutionary analyses were conducted in MEGA X.
C)
